# Admission NT-proBNP as a Prognostic Biomarker for Ventilator Weaning Failure: Implications for Tracheostomy Timing

**DOI:** 10.3390/biomedicines14040916

**Published:** 2026-04-17

**Authors:** Ah Young Leem, Shihwan Chang, Chanho Lee, Mindong Sung, Hye Young Hong, Geun In Lee, Youngmok Park, Seung Hyun Yong, Sang Hoon Lee, Song Yee Kim, Kyung Soo Chung, Eun Young Kim, Ji Ye Jung, Young Ae Kang, Moo Suk Park, Young Sam Kim, Se Hyun Kwak, Su Hwan Lee

**Affiliations:** 1 Division of Pulmonary and Critical Care Medicine, Department of Internal Medicine, Severance Hospital, Yonsei University College of Medicine, Seoul 03722, Republic of Korea; yimayoung@yuhs.ac (A.Y.L.); shihwan87@yuhs.ac (S.C.); chanholee@yuhs.ac (C.L.); mdsung@yuhs.ac (M.S.); hhy338@yuhs.ac (H.Y.H.); nerrnerr86@yuhs.ac (G.I.L.); 0mokfv@yuhs.ac (Y.P.); roneirire@yuhs.ac (S.H.Y.); cloud9@yuhs.ac (S.H.L.); dobie@yuhs.ac (S.Y.K.); chungks@yuhs.ac (K.S.C.); narae97@yuhs.ac (E.Y.K.); stopyes@yuhs.ac (J.Y.J.); mdkang@yuhs.ac (Y.A.K.); pms70@yuhs.ac (M.S.P.); ysamkim@yuhs.ac (Y.S.K.); 2 Division of Pulmonology, Allergy and Critical Care Medicine, Department of Internal Medicine, Severance Hospital, Yonsei University College of Medicine, Yongin-si 16995, Republic of Korea

**Keywords:** NT-proBNP, biomarker, ventilator weaning, tracheostomy, weaning-induced cardiac dysfunction

## Abstract

**Background/Objectives**: Ventilator weaning imposes profound hemodynamic stress, unmasking cardiopulmonary vulnerability. Since conventional predictors of post-tracheostomy weaning failure remain elusive, biomarker-driven risk stratification offers a translational approach. We evaluated the prognostic utility of admission N-terminal pro-B-type natriuretic peptide (NT-proBNP) as an early triaging tool for weaning failure and explored its therapeutic implications alongside optimal tracheostomy timing. **Methods**: In this large-scale retrospective cohort study, we analyzed 707 critically ill patients who underwent tracheostomy in a medical intensive care unit. We investigated the association between baseline NT-proBNP levels—measured as a molecular surrogate of cardiovascular stress at ICU admission; echocardiographic parameters; and weaning outcomes. Multivariable logistic regression analysis was utilized to identify independent pathophysiological predictors associated with weaning failure. **Results**: Patients experiencing weaning failure exhibited significantly elevated admission NT-proBNP levels compared to those successfully weaned (3077.0 vs. 1410.0 pg/mL, *p* < 0.001). High admission NT-proBNP (>3271 pg/mL) was independently associated with an increased risk of weaning failure (adjusted odds ratio [aOR] 2.86, 95% confidence interval [CI] 1.81–4.53, *p* < 0.001). Conversely, an early clinical intervention—tracheostomy performed within 10 days of mechanical ventilation initiation—was associated with a significantly lower risk of weaning failure (aOR 0.55, 95% CI 0.35–0.87, *p* = 0.010). Furthermore, elevated biomarker levels strongly correlated with prolonged intensive care unit stays and higher 90-day mortality. **Conclusions**: Admission NT-proBNP serves as a powerful biomarker associated with cardiopulmonary vulnerability from the earliest stages of critical illness. Integrating this diagnostic biomarker with interventional strategies like optimal tracheostomy timing has significant prognostic implications. This biomarker-guided approach facilitates personalized risk stratification from ICU admission, potentially optimizing weaning pathways for mechanically ventilated patients.

## 1. Introduction

Tracheostomy is one of the most frequently performed procedures for patients requiring prolonged mechanical ventilation in medical intensive care units (MICUs). This surgical intervention offers a multitude of clinical advantages over endotracheal intubation, including significantly improved patient comfort, easier oral hygiene, enhanced airway security, and a potential reduction in the requirement for sedative agents [1,2,3]. Despite these benefits, the transition from mechanical support to spontaneous breathing—the weaning process—remains complex and challenging. Successful liberation from mechanical ventilation following tracheostomy is not merely a procedural milestone but is a critical determinant of long-term survival and overall clinical outcomes in critically ill patients [4,5].

The physiological demands of weaning impose profound hemodynamic stress on both the respiratory and cardiovascular systems. While much focus has traditionally been on respiratory mechanics, the transition to spontaneous breathing abruptly increases venous return and left ventricular afterload, often culminating in weaning-induced cardiac dysfunction (WICD)—a primary non-respiratory etiology of weaning failure [6,7,8]. In this context, N-terminal pro-B-type natriuretic peptide (NT-proBNP), a biologically active peptide secreted by ventricular myocytes in response to increased myocardial wall stress, has emerged as a direct molecular surrogate for cardiopulmonary reserve and a promising biomarker for predicting cardiac-related weaning difficulties [9,10,11]. However, prior studies were limited by relatively small sample sizes and predominantly focused on patients admitted to the ICU in a perioperative setting.

Furthermore, the “timing” of tracheostomy—categorized as early or late—continues to be a subject of intense clinical debate. Although large-scale trials such as TracMAN have investigated its impact, previous studies have not consistently demonstrated a significant association between early tracheostomy and improved weaning outcomes, highlighting the limitations of a “one-size-fits-all” approach that fails to account for pathophysiological heterogeneity. Moreover, its relationship with cardiac biomarkers remains under-researched [3,12].

Despite the clinical importance of these factors, studies specifically focusing on the combined influence of cardiac biomarkers and tracheostomy timing on weaning outcomes in a MICU population remain limited. Therefore, this study aimed to evaluate the prognostic value of admission NT-proBNP as a predictive biomarker for ventilator weaning failure and investigate its therapeutic implications in conjunction with optimal tracheostomy timing.

## 2. Materials and Methods

### 2.1. Study Design and Population

**Figure 1:**
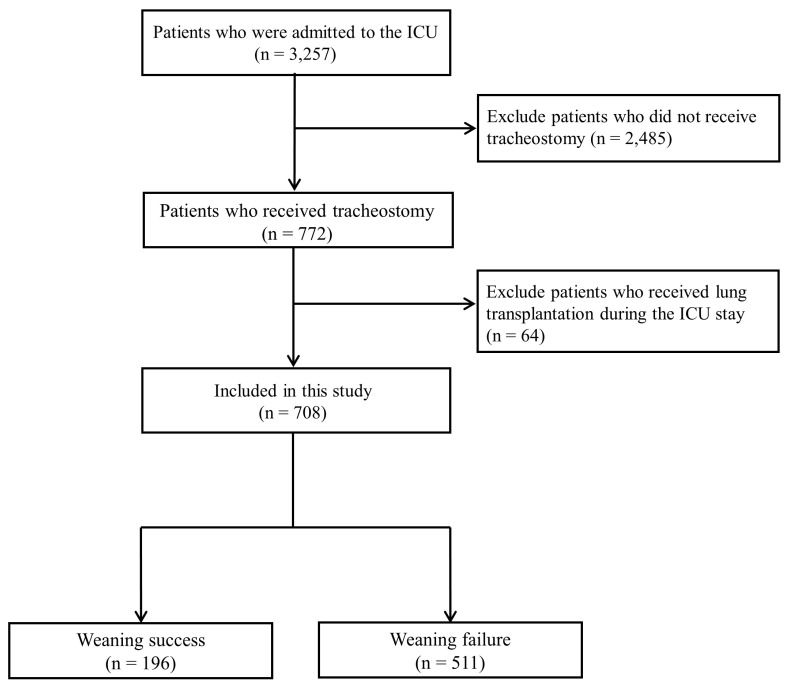
Study flow diagram.

This retrospective cohort study included adult patients who underwent tracheostomy in the MICU of a tertiary referral hospital in South Korea between July 2016 and October 2024. In all cases, surgical tracheostomies were conducted at bedside by otolaryngologists. During the study period, a total of 3257 patients were admitted to the ICU. Among them, 2485 patients who did not undergo tracheostomy during their ICU stay were excluded. Consequently, 772 patients who underwent tracheostomy were initially identified for further evaluation (Figure 1).

Patients who underwent lung transplantation during their MICU stay were excluded (n = 65), given the distinct clinical course and ventilator weaning trajectory in this population. After these exclusions, a total of 707 patients who underwent tracheostomy in the medical ICU were included in the final analysis.

Patients were subsequently categorized according to ventilator weaning outcomes into a weaning-success group (n = 196) and a weaning-failure group (n = 511). Ventilator weaning outcomes were assessed following tracheostomy and during the ICU course, as defined in Section 2.3.

### 2.2. Data Collection and Definitions

Baseline characteristics including age, sex, body mass index, and smoking history were recorded. Severity of illness at the time of MICU admission was assessed using the Acute Physiology and Chronic Health Evaluation (APACHE) II, Sequential Organ Failure Assessment (SOFA), and Simplified Acute Physiology Score II. Comorbidities were evaluated using the Charlson Comorbidity Index (CCI), along with specific histories of diabetes mellitus, malignancy, chronic kidney disease, coronary artery disease, congestive heart failure, chronic obstructive pulmonary disease, and asthma.

Clinical parameters related to tracheostomy and ventilation were also collected: reason for intubation (categorized into pulmonary, cardiac, neurologic, neuromuscular, and other causes) and tracheostomy timing (“early tracheostomy” was defined as a tracheostomy performed within 10 days of initiating mechanical ventilation, a threshold selected based on representative clinical trials [3,12] and institutional clinical protocols). For additional sensitivity analysis to ensure the robustness of the findings, a 7-day cutoff was also utilized. As this was a retrospective observational study, the exact timing of tracheostomy was not strictly protocolized; instead, the decision to perform a tracheostomy and its specific timing were determined on a case-by-case basis at the discretion of the attending intensivists. These clinical decisions were guided by the patient’s overall trajectory, including the anticipated need for prolonged mechanical ventilation, hemodynamic and respiratory stability, and the timing of obtaining informed consent from surrogate decision-makers. Laboratory findings at MICU admission as well as cardiac evaluation (baseline laboratory values (albumin, lactate, C-reactive protein, and NT-proBNP) and echocardiographic findings (ejection fraction, ratio of early diastolic mitral inflow velocity to early diastolic mitral annular tissue velocity [E/E’ ratio], and right ventricular systolic pressure)) were obtained. Ventilator weaning failure after tracheostomy was defined as the inability to achieve unassisted breathing for a minimum of 7 consecutive days during the ICU stay. This included (1) death while receiving invasive mechanical ventilation, (2) the clinical necessity to resume mechanical ventilation after a trial of spontaneous breathing, or (3) persistent dependence on invasive ventilation at the time of ICU discharge or transfer to a general ward or long-term care hospital (including conversion to home mechanical ventilation). Regarding the reviewer’s inquiry on reintubation, as all patients in this cohort already had an established surgical airway, weaning failure was characterized by the resumption of mechanical support rather than tracheal re-intubation. Weaning success was defined as successful liberation from mechanical ventilation, maintained for 7 consecutive days, or discharge from the ICU with unassisted breathing, whichever occurred first.

### 2.3. Outcome Measures

The primary outcome was successful weaning from mechanical ventilation after tracheostomy. Secondary outcomes included the length of stay in the ICU and hospital, as well as 90-day and in-hospital mortality rates.

### 2.4. Statistical Analysis

For continuous variables, Shapiro–Wilk tests were performed to determine the normality of the data distribution. Normally distributed data are presented as the mean ± standard deviation, and Student’s *t*-test was used for comparison. Non-normally distributed data are expressed as the median (25th–75th percentiles), and the Mann–Whitney U-test was employed. Specifically, given the highly skewed distribution of NT-proBNP levels, this variable was analyzed using non-parametric methods without log transformation.

Categorical variables are presented as frequencies and percentages and were compared using the chi-squared test or Fisher’s exact test. To identify independent factors associated with weaning failure, a binary multivariate logistic regression analysis was performed using the enter method. To minimize the influence of potential confounders, the model was adjusted for age, sex, BMI, disease severity (APACHE II and SOFA scores), and comorbidities (CCI, which accounts for pre-existing cardiac and renal disease). Baseline laboratory markers of systemic inflammation, such as lactate and CRP, were also considered in the initial screening for the model. Multicollinearity among the independent variables was assessed using the Variance Inflation Factor (VIF), with a VIF < 10 considered acceptable. Missing data were handled using a complete-case analysis approach. For NT-proBNP, a specific cutoff value of 3271 pg/mL was used to assess its association as a binary predictor. The optimal cutoff value for NT-proBNP was determined using the Youden index from receiver operating characteristic curve analysis. The cumulative probability of weaning success was estimated using the Kaplan–Meier method and compared between subgroups using the log-rank test.

Subgroup analyses were conducted for 90-day survivors to minimize the confounding influence of early mortality on weaning outcomes. These subgroup analyses were considered exploratory and hypothesis-generating; therefore, no formal adjustments for multiple testing were applied, and the resulting *p*-values should be interpreted with caution. Statistical significance was defined as a *p*-value < 0.05. All statistical analyses were performed using SPSS software (version 28.0, IBM Corp., Armonk, NY, USA).

To evaluate the incremental predictive value of NT-proBNP beyond existing clinical severity scores, we constructed two logistic regression models: a base model comprising SOFA and APACHE II scores alone (Model 1), and an extended model additionally incorporating NT-proBNP > 3271 pg/mL (Model 2). The discriminative performance of each model was quantified by the area under the receiver operating characteristic curve (AUC), and the statistical significance of the difference in AUC between models was assessed using the DeLong test. The Integrated Discrimination Improvement (IDI) was also calculated to quantify the net improvement in predicted probability discrimination attributable to the addition of NT-proBNP.

## 3. Results

### 3.1. Baseline Patient Characteristics

A total of 707 patients who underwent tracheostomy in the MICU were included in the analysis, of whom 196 (27.7%) achieved successful ventilator weaning and 511 (72.3%) experienced weaning failure. Baseline characteristics according to weaning outcome are summarized in Table 1.

We observed several baseline imbalances between the two groups. There were no significant differences between the two groups in terms of age, sex, body mass index, smoking history, or major comorbidities such as diabetes mellitus, chronic kidney disease, chronic obstructive pulmonary disease, or asthma. However, patients in the weaning-failure group had a significantly higher CCI compared with those in the weaning-success group (3.5 ± 2.4 vs. 2.9 ± 2.2, *p* = 0.003). Malignancy (37.0% vs. 25.5%, *p* = 0.004) and coronary artery disease (20.9% vs. 11.2%, *p* = 0.003) were also more prevalent in the weaning-failure group. Furthermore, severity of illness at the time of MICU admission was significantly greater in the weaning-failure group, as reflected by higher scores for SOFA (9.2 ± 3.8 vs. 8.2 ± 3.5, *p* = 0.003), APACHE II (27.5 ± 8.1 vs. 25.1 ± 7.3, *p* < 0.001), and Simplified Acute Physiology Score II (50.2 ± 17.2 vs. 46.1 ± 16.1, *p* = 0.004). 

The reason for intubation did not differ significantly between groups. However, the duration of mechanical ventilation before tracheostomy was longer in patients with weaning failure than in those with weaning success (14.0 [11.0–18.0] days vs. 12.0 [7.0–16.0] days, *p* = 0.048).

Early tracheostomy was more frequently performed in the weaning-success group, both within 10 days (36.2% vs. 24.0%, *p* = 0.002) and within 7 days (23.0% vs. 15.2%, *p* = 0.022).

Severity of illness at the time of MICU admission was significantly greater in the weaning-failure group, as reflected by higher SOFA (9.2 ± 3.8 vs. 8.2 ± 3.5, *p* = 0.003), APACHE II (27.5 ± 8.1 vs. 25.1 ± 7.3, *p* < 0.001), and Simplified Acute Physiology Score II scores (50.2 ± 17.2 vs. 46.1 ± 16.1, *p* = 0.004). Echocardiographic parameters, including left ventricular ejection fraction, E/E′ ratio, and right ventricular systolic pressure, did not differ significantly between the groups. Among laboratory findings, NT-proBNP levels were significantly higher in the weaning-failure group (3077.0 [686.3–9257.3] pg/mL vs. 1410.0 [442.0–4742.0] pg/mL, *p* < 0.001), whereas albumin, lactate, and C-reactive protein levels were comparable. In the ROC curve analysis for predicting weaning failure, admission NT-proBNP yielded an AUC of 0.596 (95% CI, 0.556–0.636). At the optimal cutoff of 3271 pg/mL, the sensitivity and specificity were 48.1% and 72.4%, respectively.

### 3.2. Clinical Outcomes of Ventilator Weaning

Clinical outcomes according to weaning status are presented in Table 2. ICU length of stay was significantly longer in patients with weaning failure compared with those who achieved successful weaning (34.0 [22.0–50.0] vs. 21.0 [14.0–37.0] days, *p* = 0.001), whereas the hospital length of stay was significantly longer in patients with weaning success than in those with weaning failure (73.5 [49.0–117.3] vs. 66.0 [40.0–111.0] days, *p* < 0.001). Both 90-day mortality (54.5% vs. 3.1%, *p* < 0.001) and in-hospital mortality (67.7% vs. 7.1%, *p* < 0.001) were markedly higher in the weaning-failure group.

### 3.3. Factors Associated with Weaning Failure

Multivariate logistic regression analysis, adjusting for demographics, severity of illness, and underlying comorbidities (including cardiac and renal disease via CCI), identified several independent factors associated with weaning failure after tracheostomy (Table 3). The presence of malignancy (adjusted odds ratio [OR] 1.82, 95% confidence interval [CI] 1.14–2.91, *p* = 0.012) and elevated NT-proBNP levels (>3271 pg/mL; adjusted OR 2.86, 95% CI 1.81–4.53, *p* < 0.001) was independently associated with an increased likelihood of weaning failure. In contrast, early tracheostomy performed within 10 days of mechanical ventilation initiation was independently linked to a lower likelihood of weaning failure (adjusted OR 0.55, 95% CI 0.35–0.87, *p* = 0.010). Age, sex, body mass index, CCI, APACHE II score, and SOFA score were not independently associated with weaning outcomes.

To formally assess the incremental predictive value of NT-proBNP beyond established clinical severity scores, we compared the discriminative performance of two logistic regression models in 522 patients with complete data. The base model incorporating SOFA and APACHE II alone yielded an AUC of 0.582 (95% CI: 0.528–0.636). The addition of NT-proBNP > 3271 pg/mL significantly improved the AUC to 0.642 (95% CI: 0.591–0.693), representing a statistically significant increment of ΔAUC = +0.060 (DeLong test: *p* = 0.011). The Integrated Discrimination Improvement (IDI) was 0.030, confirming that NT-proBNP meaningfully enhanced the model’s ability to discriminate between patients with and without weaning failure beyond what is captured by severity-of-illness scores alone (Supplementary Figure S1).

### 3.4. Cumulative Weaning Probability According to NT-proBNP Levels and Tracheostomy Timing

**Figure 2:**
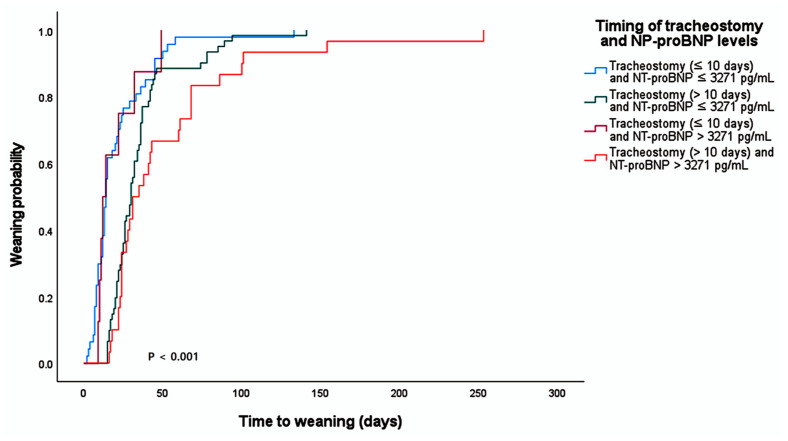
**Cumulative probability of weaning success stratified by NT-proBNP levels and tracheostomy timing.** The Kaplan–Meier curves represent the cumulative incidence of successful weaning. Group comparisons were performed using the log-rank test.

To examine the combined associations of cardiac stress markers and procedural timing, patients were stratified into four groups based on the identified independent predictors of NT-proBNP levels (cutoff: 3271 pg/mL) and tracheostomy timing (cutoff: 10 days). Kaplan–Meier analysis revealed a significant difference in the cumulative probability of weaning success among the four groups (log-rank *p* < 0.001; Figure 2). The highest weaning success rate was observed in the group with both low NT-proBNP levels and early tracheostomy (≤10 days). Conversely, patients with both elevated NT-proBNP levels (>3271 pg/mL) and tracheostomy timing (>10 days) showed the lowest probability of successful weaning from mechanical ventilation. Patients meeting only one favorable criterion (either early tracheostomy or low NT-proBNP) demonstrated intermediate weaning trajectories. These findings suggest that the integration of cardiovascular reserve assessment and optimal timing of tracheostomy is clinically relevant for evaluating weaning outcomes in the MICU.

### 3.5. Analysis of the Time to Ventilator Weaning Among Patients with Successful Weaning

In this study, a total of 196 patients achieved successful ventilator weaning. Among these patients, the duration from initiation of mechanical ventilation to successful weaning was 25.0 (16.0–40.5) days. Among patients who achieved successful ventilator weaning, the time from initiation of mechanical ventilation to successful weaning was significantly longer in those with elevated NT-proBNP levels than in those without elevated NT-proBNP levels (31.0 [22.0–62.3] vs. 24.0 [15.0–36.0] days, *p* = 0.007).

In addition, patients who underwent early tracheostomy (within 10 days) showed a longer duration from mechanical ventilation initiation to successful weaning compared with those who underwent late tracheostomy (30.0 [22.0–42.0] vs. 14.0 [9.0–29.8] days, *p* < 0.001).

### 3.6. Sensitivity Analyses

Subgroup analyses restricted to 90-day survivors (n = 421) yielded consistent findings (Supplementary Tables S1–S3). In this subgroup, NT-proBNP levels (*p* = 0.033) and early tracheostomy remained significantly associated with successful weaning (*p* = 0.007). Multivariate analysis for this subgroup confirmed that NT-proBNP > 3271 pg/mL (OR 2.254, *p* = 0.001) and late tracheostomy (OR for early tracheostomy 0.474, *p* = 0.006) were consistent independent predictors.

Among the 708 patients admitted to the MICU for mechanical ventilation, seven were admitted for exacerbation of neuromuscular diseases; notably, all of them failed to be weaned from mechanical ventilation. After excluding these seven individuals, a secondary analysis was performed on the remaining 701 patients. The results regarding baseline characteristics, clinical weaning outcomes, and risk factors for weaning failure showed no significant differences from the findings presented in Table 1, Table 2 and Table 3 (Supplementary Tables S4–S6).

## 4. Discussion

In this large cohort of critically ill patients who underwent tracheostomy in a MICU, we identified several clinically relevant predictors associated with ventilator weaning failure. The principal findings of this study are that admission NT-proBNP serves as a powerful prognostic biomarker and that early tracheostomy is associated with a significantly higher likelihood of successful weaning. These associations remained consistent across clinically important subgroups, including 90-day survivors. Collectively, these findings highlight the clinical utility of assessing cardiovascular stress at the molecular level and integrating it with procedural timing to optimize weaning outcomes.

Weaning from mechanical ventilation represents a critical physiological transition characterized by abrupt increases in respiratory and cardiovascular workload [6,7,8,13]. While respiratory mechanics have traditionally dominated the clinical assessment of weaning readiness, accumulating evidence suggests that weaning-induced cardiac dysfunction (WICD)—particularly left ventricular diastolic impairment and increased myocardial wall stress— is a pivotal factor associated with weaning failure [6,7,8,13].

Our findings align with prior research demonstrating that elevated natriuretic peptides reflect subclinical or overt cardiac dysfunction during spontaneous breathing trials and are associated with weaning-induced cardiopulmonary decompensation. In small observational cohorts, NT-proBNP levels at the end of SBT were significantly higher in patients who were unsuccessful in weaning compared with those who succeeded, supporting the role of NT-proBNP as a marker of myocardial stress and a predictor of weaning failure [11,14]. Moreover, a recent cohort study suggests that changes in NT-proBNP levels may provide additional predictive value for weaning outcomes [10]. While most previous studies focused on NT-proBNP levels measured during spontaneous breathing trials or immediately before extubation, our study uniquely demonstrates the utility of baseline NT-proBNP measured at the time of ICU admission. This highlights its value as an early risk-stratification tool, allowing clinicians to identify “cardiopulmonary vulnerability” from the very beginning of an ICU stay and plan long-term airway management weeks before weaning begins. Although the standalone predictive performance of admission NT-proBNP was modest (AUC 0.596, 95% CI 0.556–0.636), its clinical significance is underscored by its role as a robust independent predictor (aOR 2.86) even after adjusting for disease severity and comorbidities. Importantly, a formal comparative model analysis demonstrated that NT-proBNP provides significant incremental predictive value beyond SOFA and APACHE II scores: the addition of NT-proBNP > 3271 pg/mL to a base model comprising these two severity scores significantly improved the AUC from 0.582 (95% CI: 0.528–0.636) to 0.642 (95% CI: 0.591–0.693) (ΔAUC + 0.060, DeLong test *p* = 0.011), with an IDI of 0.030. Notably, SOFA and APACHE II themselves did not reach independent significance in multivariable analysis (*p* = 0.737 and *p* = 0.470, respectively), whereas NT-proBNP remained a highly significant predictor (*p* < 0.001), suggesting that it captures a dimension of cardiopulmonary vulnerability not adequately reflected by general severity-of-illness scores alone. Specifically, the relatively high specificity (72.4%) at the optimal cutoff of 3271 pg/mL suggests that elevated admission NT-proBNP effectively identifies a high-risk phenotype predisposed to weaning failure. This allows for a longer clinical window to optimize cardiovascular status before proceeding to tracheostomy and weaning.

Notably, echocardiographic variables such as LVEF, E/E’ ratio, and RVSP did not differ significantly between the groups. This discrepancy likely reflects the inherent limitations of resting echocardiography, which provides only a static morphological snapshot that may fail to capture the dynamic cardiovascular stress induced by weaning. In contrast, NT-proBNP serves as a sensitive molecular integrator of cumulative myocardial wall stress over time. It may more effectively reflect subclinical diastolic dysfunction or impaired cardiovascular reserve—often termed ‘weaning-induced cardiac dysfunction’—that remains latent during resting imaging but manifests during the physiological challenge of spontaneous breathing. Furthermore, worsening pulmonary dynamics, such as hypoxia, can trigger pulmonary vasoconstriction and elevate pulmonary artery pressure. This increased right ventricular afterload, through ventricular interdependence, exacerbates myocardial wall stress and contributes to elevated NT-proBNP levels, suggesting that the marker reflects complex pulmonary–cardiac interactions during weaning. These findings support the concept that biochemical markers can overcome the limitations of intermittent imaging, providing a more continuous and objective molecular surrogate for cardiovascular reserve in critically ill patients with evolving hemodynamics.

The timing of tracheostomy remains an area of ongoing debate. Research on patients with coronavirus disease 2019 also showed no significant differences between the early and late tracheostomy groups in terms of overall mortality or the time to successful liberation from mechanical ventilation [15]. Furthermore, large randomized trials, including the TracMan study, did not demonstrate a consistent survival or ventilator liberation benefit associated with early tracheostomy [3]. In the TracMan study, early tracheostomy (≤4 days) versus late tracheostomy (≥10 days) did not significantly reduce 30-day mortality or the number of ventilator days. Similarly, systematic reviews and meta-analyses have reported heterogeneous results regarding the impact of tracheostomy timing on ICU outcomes, suggesting that optimal timing remains unclear [12]. We postulate that these conflicting results in the previous literature may stem from a “one-size-fits-all” approach that fails to account for the underlying pathophysiological heterogeneity of critically ill patients. However, most of these studies enrolled mixed ICU populations or focused predominantly on surgical or trauma patients, limiting their applicability to the MICU setting. Retrospective cohort studies in mixed ICU cohorts have yielded conflicting findings, with some suggesting that earlier tracheostomy may shorten ventilator duration but without consistent improvements in mortality. In contrast, the present study specifically evaluated a MICU cohort and demonstrated that tracheostomy performed within 10 days of mechanical ventilation initiation was independently associated with improved weaning outcomes. This 10-day threshold was selected as it represents a common clinical transition point for long-term airway management and aligns with several influential trials and meta-analyses that delineate early versus late intervention [3,12]. Furthermore, to ensure that our findings were not dependent on an arbitrary cutoff, we performed sensitivity analyses using an alternative 7-day threshold, which yielded consistent and robust results. This stability across different time points (7 and 10 days) suggests that the observed clinical trend reflects a genuine physiological advantage rather than an artifact of the chosen definition. Crucially, this study moves beyond mere prediction by integrating molecular biomarkers with procedural timing, representing a novel conceptual contribution that distinguishes it from prior literature focused solely on NT-proBNP as a weaning predictor. Our data suggests that the clinical benefit of early tracheostomy is not uniform but is closely linked to the patient’s underlying cardiovascular reserve. Our findings suggest a conceptual shift away from a “one-size-fits-all” approach toward a more individualized framework for evaluating physiological readiness for tracheostomy. However, we strictly acknowledge that translating this concept into clinical practice to actively guide procedural timing remains premature and requires prospective validation.

Several mechanisms may explain the association between early tracheostomy and weaning success observed in our cohort. Early tracheostomy is linked to potential reductions in sedation requirements, improved patient comfort, enhanced airway clearance, and earlier initiation of active rehabilitation—all of which may contribute to more efficient ventilator liberation. Importantly, when considered alongside NT-proBNP levels, our findings suggest that procedural timing alone may be insufficient to evaluate weaning success; instead, the interaction between this therapeutic intervention and underlying cardiovascular reserve appears to be crucial.

From a translational and interdisciplinary perspective, the combined assessment of tracheostomy timing and cardiac biomarkers offers a more nuanced framework for individualized weaning strategies. Specifically, we propose a clinical decision framework consisting of: (1) identifying a ‘high-risk phenotype’ using admission NT-proBNP (>3271 pg/mL); (2) implementing targeted cardiovascular optimization; and (3) considering a proactive tracheostomy strategy—ideally within 10 days—to enhance airway clearance and reduce sedation. This integrated approach, combining molecular risk stratification with optimal procedural timing, offers a more individualized pathway toward successful ventilator liberation than a standard ‘one-size-fits-all’ strategy. This study adds to the literature by shifting the paradigm from simple prognosis to proactive clinical strategy. In contrast, in patients with preserved cardiac reserve, earlier tracheostomy may be associated with meaningful advantages in supporting ventilator liberation without imposing overwhelming hemodynamic stress. Ultimately, these findings offer a hypothesis-generating step toward “precision weaning” in the MICU. However, rigorous prospective validation is required before molecular profiles can be routinely used to tailor the timing of invasive procedures.

This study possesses several distinct strengths. First, it is a large-scale investigation involving more than 700 patients, providing robust data and statistical power to validate the diagnostic and prognostic utility of this biomarker for analyzing weaning outcomes and their associated risk factors in patients undergoing tracheostomy. Second, being a single-center study allowed for a consistent weaning protocol to be applied by a dedicated team of intensification and critical care specialists, ensuring standardized clinical management. Furthermore, by focusing specifically on medical ICU patients and excluding lung transplant recipients, we were able to evaluate a more homogeneous patient population compared to previous studies, thereby enhancing the internal validity of our pathophysiological findings.

This study has several limitations. First, its retrospective design precludes causal inference, and residual confounding cannot be fully excluded despite multivariable adjustment. Second, we acknowledge the presence of selection and survivorship bias, as our cohort only included patients who survived long enough to undergo tracheostomy and excluded those who were successfully extubated early. To mitigate the potential impact of these biases, we performed a sensitivity analysis restricted to 90-day survivors, which yielded consistent results and supports the robustness of our observations. Furthermore, the association between early tracheostomy and weaning success persisted in older adult patients, suggesting that the observed benefit was not merely driven by survivorship bias. Third, our subgroup analyses may be underpowered due to the reduced sample sizes within specific categories. Because we did not adjust for multiple comparisons, there is an inherent risk of false-positive findings. Consequently, the results of the subgroup analyses should be viewed as exploratory, and further large-scale studies are warranted to confirm these specific associations. Fourth, the interpretation of NT-proBNP levels can be confounded by renal dysfunction, as impaired renal clearance can lead to elevated circulating levels independent of acute cardiovascular stress. Although there was no significant difference in the baseline prevalence of chronic kidney disease between the two groups, and we adjusted for baseline comorbidities utilizing the CCI, our retrospective design precluded a detailed analysis of dynamic renal function changes, such as acute kidney injury, during ICU stays. Fifth, specific ventilatory parameters such as PEEP at the time of tracheostomy were unavailable. While high PEEP can theoretically increase myocardial wall stress and confound NT-proBNP levels, baseline P/F ratios were comparable between groups, and all procedures followed a standardized protocol requiring respiratory stability (typically PEEP ≤ 10 cmH_2_O), likely minimizing this impact. Finally, NT-proBNP measurements were obtained at baseline and may not have fully captured dynamic molecular changes during the weaning process. Additionally, data were derived from a single tertiary center, which may limit generalizability. This study was conducted at one of the largest tertiary referral hospitals in Korea with a large number of dedicated intensivists; therefore, the high severity of illness among patients in the ICU may have influenced the results. Nonetheless, the large sample size, inclusion of a broad MICU population, and robust subgroup analyses strengthen the validity of the observed associations.

## 5. Conclusions

In conclusion, this study demonstrated that elevated admission NT-proBNP levels and delayed tracheostomy were independently associated with ventilator weaning failure in MICU patients. While these findings highlight the potential value of integrating molecular cardiovascular biomarkers with interdisciplinary procedural decision-making, utilizing these markers to actively guide the timing of tracheostomy remains premature. The results of this retrospective study should be interpreted as hypothesis-generating. Future prospective cohort studies and randomized controlled trials are essential to validate these results and to establish the true clinical applicability of biomarker-guided strategies for personalized ventilator weaning in critically ill patients.

## Figures and Tables

**Table 1:** Baseline characteristics of weaning-success and -failure patients.

Variables	Weaning		*p*-Value
	Success	Failure	
	(N = 196)	(N = 511)	
Age (years)	66.2 ± 14.4	67.0 ± 14.0	0.505
≥65 years, n (%)	110 (56.1)	320 (62.6)	
<65 years, n (%)	86 (43.9)	191 (37.4)	
Sex			0.304
Male, n (%)	132 (67.3)	323 (63.2)	
Female, n (%)	64 (32.7)	188 (36.8)	
BMI (kg/m^2^)	22.7 ± 7.4	22.3 ± 5.1	0.331
Smoking history, n (%)	60 (30.6)	180 (35.3)	0.240
Comorbidities			
CCI	2.9 ± 2.2	3.5 ± 2.4	0.003
DM, n (%)	73 (37.2)	201 (39.3)	0.610
Malignancy, n (%)	50 (25.5)	189 (37.0)	0.004
CKD, n (%)	37 (18.9)	130 (25.4)	0.066
CAD, n (%)	22 (11.2)	107 (20.9)	0.003
CHF, n (%)	17 (8.7)	70 (13.7)	0.074
COPD, n (%)	32 (16.3)	76 (14.9)	0.631
Asthma, n (%)	16 (8.2)	34 (6.7)	0.513
Primary reason for intubation			0.163
Pulmonary problems, n (%)	150 (76.5)	420 (82.2)	
Cardiac problems, n (%)	15 (7.7)	30 (5.9)	
Neurologic problems, n (%)	17 (9.7)	29 (5.7)	
Neuromuscular problems, n (%)	0	7 (1.4)	
Others, n (%)	14 (7.1)	24 (4.7)	
MV before tracheostomy (days)	12.0 (7.0–16.0)	14.0 (11.0–18.0)	0.048
Tracheostomy performed within 10 days, n (%)	63 (36.2)	107 (24.0)	0.002
Tracheostomy performed within 7 days, n (%)	40 (23.0)	68 (15.2)	0.022
Duration of MV prior to successful weaning (days)	25.0 (16.0–40.5)		
SOFA score	8.2 ± 3.5	9.2 ± 3.8	0.003
APACHE II score	25.1 ± 7.3	27.5 ± 8.1	<0.001
SAPS II	46.1 ± 16.1	50.2 ± 17.2	0.004
Echocardiographic findings			
EF (%)	61.8 ± 12.1	59.8 ± 14.1	0.078
E/E’ ratio	12.7 ± 8.1	13.4 ± 5.7	0.265
RVSP (mmHg)	38.2 ± 12.9	39.9 ± 14.1	0.189
Laboratory findings			
Albumin (g/dL)	2.8 ± 0.5	2.8 ± 0.4	0.782
Lactate (mmol/L)	1.7 (1.3–2.5)	2.1 (1.3–3.6)	0.267
CRP (mg/L)	126.6 ± 106.2	108.8 ± 89.8	0.114
NT-proBNP (pg/mL)	1410.0 (442.0–4742.0)	3077.0 (686.3–9257.3)	<0.001

Note: Continuous variables are presented as mean ± standard deviation (SD) or median (interquartile range [IQR]). Categorical variables are presented as number (%). Abbreviations: BMI, body mass index; CCI, Charlson Comorbidity Index; DM, diabetes mellitus; CKD, chronic kidney disease; CAD, coronary artery disease; CHF, congestive heart failure; COPD, chronic obstructive pulmonary disease; MV, mechanical ventilation; SOFA, Sequential Organ Failure Assessment; APACHE, Acute Physiology and Chronic Health Evaluation; SAPS, Simplified Acute Physiology Score; CRP, C-reactive protein; EF, ejection fraction; RVSP, right ventricular systolic pressure; NT-proBNP, N-terminal pro-B-type natriuretic peptide.

**Table 2:** Clinical outcomes of weaning-success and -failure patients.

Variables	Weaning		*p*-Value
	Success	Failure	
	(N = 196)	(N = 511)	
Hospital LOS (days)	73.5 (49.0–117.3)	66.0 (40.0–111.0)	<0.001
ICU LOS (days)	21.0 (14.0–37.0)	34.0 (22.0–50.0)	0.001
90-day mortality, n (%)	6 (3.1)	278 (54.5)	<0.001
In-hospital mortality, n (%)	14 (7.1)	346 (67.7)	<0.001

Note: Continuous variables are presented as median (interquartile range [IQR]). Categorical variables are presented as number (%). Abbreviations: ICU, intensive care unit; LOS, length of stay.

**Table 3:** Factors independently associated with weaning failure after tracheostomy.

Variable	Adjusted		
	OR	95% CI	*p*-Value
Age	0.99	0.98–1.01	0.374
Female sex	1.17	0.74–1.87	0.502
BMI (kg/m^2^)	0.97	0.94–1.01	0.115
CCI	0.99	0.88–1.11	0.835
Malignancy	1.82	1.14–2.91	0.012
CAD	1.67	0.94–2.96	0.079
APACHE II	1.01	0.98–1.04	0.470
SOFA	0.99	0.93–1.06	0.737
NT-proBNP > 3271 pg/mL	2.86	1.81–4.53	<0.001
Early tracheostomy (≤10 days)	0.55	0.35–0.87	0.010

Note: Analysis was conducted using multivariate logistic regression, adjusted for age, sex, body mass index, APACHE II score, SOFA score, and Charlson Comorbidity Index (which accounts for pre-existing cardiac and renal diseases). Abbreviations: BMI, body-mass index; CCI, Charlson Comorbidity Index; CAD, coronary artery disease; APACHE, the Acute Physiology and Chronic Health Evaluation; SOFA, Sequential Organ Failure Assessment; NT-proBNP, N-terminal pro-B-type natriuretic peptide.

## Data Availability

The complete data set used in this study will be made available to researchers upon request addressed to the corresponding author.

## Supplementary Materials

The following supporting information can be downloaded at: https://www.mdpi.com/article/10.3390/biomedicines14040916/s1, Supplementary Table S1: Baseline characteristics of weaning-success and -failure patients (90-day Survivors); Supplementary Table S2: Clinical outcomes of weaning-success and -failure patients (90-day Survivors); Supplementary Table S3: Independent risk factors for weaning failure after tracheostomy (90-day Survivors); Supplementary Table S4: Baseline characteristics of weaning-success and -failure patients excluding those with neuromuscular diseases; Supplementary Table S5: Clinical outcomes of weaning-success and -failure patients excluding those with neuromuscular diseases; Supplementary Table S6: Independent risk factors for weaning failure after tracheostomy patients excluding those with neuromuscular diseases; Supplementary Figure S1: Receiver operating characteristic (ROC) curves comparing the incremental predictive value of NT-proBNP for ventilator weaning failure.

## Abbreviations

The following abbreviations are used in this manuscript:

MICU	Medical intensive care unit
APACHE	Acute Physiology and Chronic Health Evaluation
SOFA	Sequential Organ Failure Assessment
CCI	Charlson Comorbidity Index
OR	Odds ratio
CI	Confidence interval
