# Supplementary material for: Admission NT-proBNP as a Prognostic Biomarker for Ventilator Weaning Failure: Implications for Tracheostomy Timing
Source: Biomedicines. 2026 Apr 17;14(4):916. doi: 10.3390/biomedicines14040916 (PMC13113426; doi:10.3390/biomedicines14040916)
Supplement: Supplementary file 1 [file biomedicines-14-00916-s001.zip › Supplementary Tables.pdf]

Supplementary Table 1. Baseline characteristics of weaning success and failure patients (90-day Survivors)

Variables	Weaning		*P*-value
	Success (N=189)	Failure (N=232)	
**Age, years**	66.3 ± 14.5	66.5 ± 15.4	0.909
**Sex**			0.538
**Male**	126 (66.7)	148 (63.8)	
**Female**	63 (33.3)	84 (36.2)	
**BMI (kg/m2)**	22.8 ± 7.5	22.1 ± 5.9	0.246
**Smoking history**	58 (30.7)	76 (32.8)	0.650
**Comorbidities**			
**CCI**	2.9 ± 2.2	3.2 ± 2.0	0.153
**DM**	71 (37.6)	98 (42.2)	0.330
**Malignancy**	47 (24.9)	63 (27.2)	0.595
**CKD**	37 (19.6)	62 (26.7)	0.085
**CAD**	21 (11.1)	48 (20.7)	0.008
**CHF**	15 (7.9)	36 (15.5)	0.024
**COPD**	31 (16.4)	40 (17.2)	0.819
**Asthma**	16 (8.5)	13 (5.6)	0.249
**Primary reason for intubation**			0.104
**Pulmonary problems**	144 (76.2)	175 (75.4)	
**Cardiac problems**	15 (7.9)	23 (9.9)	
**Neurologic problems**	17 (9.0)	17 (7.3)	
**Neuromuscular problems**	0	7 (3.0)	
**Others**	13 (6.9)	9 (3.9)	
**MV before tracheostomy, days**	13.0 (8.0 - 16.0)	14.0 (11.0 - 18.0)	0.089
**Tracheostomy performed within 10 days (n = 113)**	63 (37.5)	50 (24.5)	0.007
**Tracheostomy performed within 7 days (n = 71)**	40 (23.8)	31 (15.2)	0.035
**Duration of MV prior to tracheostomy (days)**	25.0 (16.0 - 41.0)		
**Duration from tracheostomy to weaning (days)**	10.0 (4.0 - 24.0)		
**SOFA**	8.2 ± 3.6	8.6 ± 3.5	0.270
**APACHE II**	25.1 ± 7.3	26.8 ± 8.1	0.033
**SAPS II**	46.0 ± 16.1	50.2 ± 15.9	0.009
**Echocardiographic findings**			

**EF (%)**	61.6 ± 12.1	60.1 ± 14.1	0.285
**E/E’**	12.8 ± 8.2	13.1 ± 5.3	0.691
**RVSP (mmHg)**	38.3 ± 12.9	39.8 ± 13.9	0.308
**Laboratory findings**			
**Albumin (g/dL)**	2.7 ± 0.5	2.8 ± 0.5	0.725
**Lactate (mmol/L)**	3.6 ± 12.7	2.9 ± 2.9	0.464
**CRP (mg/L)**	123.7 ± 101.7	102.5 ± 85.9	0.052
**NT-proBNP (pg/mL)**	1300.5 (444.3 - 4676.5)	2439.0 (555.1 - 7507.5)	0.033

Note: Continuous variables are presented as mean ± standard deviation (SD) or median (interquartile range [IQR]). Categorical variables are presented as number (%).

Abbreviations: BMI, body-mass index; CCI, charlson comorbidity index; DM, diabetes mellitus; CKD, chronic kidney disease; CAD, coronary artery disease; CHF, congestive heart failure; COPD, chronic obstructive lung disease; ROSC, return of spontaneous circulation; MV, mechanical ventilation; SOFA, sequential organ failure assessment; APACHE, the acute physiology and chronic health evaluation; SAPS, simplified acute physiology score; CRP, c-reactive protein; EF, ejection fraction; RVSP, right ventricular systolic pressure; NT-proBNP, N-terminal pro-B-type Natriuretic Peptide

Supplementary Table 2. Clinical outcomes of weaning success and failure patients (90-day Survivors)

Variables	Weaning		*P*-value
	**Success (N=196)**	**Failure (N=511)**	
**Hospital LOS (days)**	81.5 (54.5 - 123.8)	106.0 (60.0 - 167.5)	0.007
**ICU LOS (days)**	23.0 (15.0 - 37.0)	34.0 (21.0 - 56.0)	<0.001
**In-hospital mortality, n (%)**	8 (4.2)	70 (30.2)	<0.001

Note: Continuous variables are presented as median (interquartile range [IQR]). Categorical variables are presented as number (%).
Abbreviations: ICU, intensive care unit; LOS, length of stay

Supplementary Table 3. Independent risk factors for weaning failure after tracheostomy (90-day Survivors)

Variable	Adjusted		
	OR	95% CI	*P*
**Age**	0.988	0.972 - 1.005	0.177
**Sex, female**	1.163	0.695 - 1.947	0.565
**Female sex**	0.964	0.925 - 1.005	0.084
**CCI**	1.004	0.890 - 1.132	0.884
**CAD**	1.52	0.808 - 2.860	0.194
**APACHE II**	1.005	0.973 - 1.038	0.747
**NT-proBNP > 3,271 pg/mL**	2.254	1.365 - 3.724	0.001
**Early tracheostomy (≤ 10 days)**	0.474	0.278 - 0.807	0.006

Note: Analyzed using multivariate logistic regression

Abbreviations: BMI, body-mass index; CCI, charlson comorbidity index; CAD, coronary artery disease; APACHE, the acute physiology and chronic health evaluation; SOFA, sequential organ failure assessment; NT-proBNP, N-terminal pro-B-type Natriuretic Peptide

Supplementary Table 4. Baseline characteristics of weaning success and failure patients excluding those with neuromuscular diseases

Variables	Weaning		*P*-value
	Success (N=196)	Failure (N=504)	
**Age, years**	66.2 ± 14.4	67.0 ± 14.0	0.478
**≥ 65 years**	110 (56.1)	316 (62.8)	0.103
**< 65 years**	86 (43.9)	187 (37.2)	
**Sex**			0.330
**Male**	132 (67.3)	319 (63.4)	
**Female**	64 (32.7)	184 (36.6)	
**BMI (kg/m2)**	22.7 ± 7.4	22.3 ± 5.1	0.376
**Smoking history**	60 (30.6)	179 (35.7)	0.207
**Comorbidities**			
**CCI**	2.9 ± 2.2	3.5 ± 2.4	0.002
**DM**	73 (37.2)	198 (39.4)	0.606
**Malignancy**	50 (25.5)	188 (37.4)	0.003
**CKD**	37 (18.9)	129 (25.6)	0.059
**CAD**	22 (11.2)	105 (20.9)	0.003
**CHF**	17 (8.7)	70 (13.9)	0.059
**COPD**	32 (16.3)	75 (14.9)	0.640
**Asthma**	16 (8.2)	34 (6.8)	0.518
**Primary reason for intubation**			0.192
**Pulmonary problems**	150 (76.5)	420 (83.5)	
**Cardiac problems**	15 (7.7)	30 (6.0)	
**Neurologic problems**	17 (8.7)	29 (5.8)	
**Others**	14 (7.1)	24 (4.8)	
**MV before tracheostomy (days)**	12.0 (7.0 - 16.0)	15.0 (11.0 - 19.0)	0.003
**Tracheostomy performed within 10 days**	63 (36.2)	102 (23.3)	0.001
**Tracheostomy performed within 7 days**	40 (23.0)	64 (14.6)	0.013
**Duration of MV prior to tracheostomy (days)**	26.0 (15.0 - 42.0)		
**Duration from tracheostomy to weaning (days)**	10.5 (4.0 - 24.5)		
**SOFA**	8.2 ± 3.5	9.2 ± 3.8	0.002
**APACHE II**	25.1 ± 7.3	27.6 ± 8.0	<0.001
**SAPS II**	46.1 ± 16.1	50.3 ± 17.3	0.003
**Echocardiographic findings**			
**EF (%)**	61.8 ± 12.1	59.7 ± 14.1	0.070

**E/E'**	12.7 ± 8.1	13.4 ± 5.7	0.256
**RVSP (mmHg)**	38.2 ± 12.9	39.8 ± 14.1	0.203
**Laboratory findings**			
**Albumin (g/dL)**	2.8 ± 0.5	2.7 ± 0.5	0.636
**Lactate (mmol/L)**	1.7 (1.3 - 2.5)	2.1 (1.4 - 3.7)	0.522
**CRP (mg/L)**	126.6 ± 106.2	109.9 ± 89.9	0.073
**NT-proBNP (pg/mL)**	1410.0 (442.0 - 4742.0)	3346.0 (879.0 - 12108.0)	<0.001

Note: Continuous variables are presented as mean ± standard deviation (SD) or median (interquartile range [IQR]). Categorical variables are presented as number (%).

Abbreviations: BMI, body-mass index; CCI, charlson comorbidity index; DM, diabetes mellitus; CKD, chronic kidney disease; CAD, coronary artery disease; CHF, congestive heart failure; COPD, chronic obstructive lung disease; ROSC, return of spontaneous circulation; MV, mechanical ventilation; SOFA, sequential organ failure assessment; APACHE, the acute physiology and chronic health evaluation; SAPS, simplified acute physiology score; CRP, c-reactive protein; EF, ejection fraction; RVSP, right ventricular systolic pressure; NT-proBNP, N-terminal pro-B-type Natriuretic Peptide

Supplementary Table 5. Clinical outcomes of weaning success and failure patients excluding those with neuromuscular diseases

Variables	Weaning		*P*-value
	**Success (N=196)**	**Failure (N=504)**	
**Hospital LOS (days)**	73.5 (49.0 - 117.3)	69.0 (40.0 - 121.0)	<0.001
**ICU LOS (days)**	21.0 (14.0 - 37.0)	34.0 (23.0 - 51.0)	<0.001
**90-day mortality, n (%)**	6 (3.1)	278 (55.4)	<0.001
**In-hospital mortality, n (%)**	14 (7.1)	345 (68.6)	<0.001

Note: Continuous variables are presented as (interquartile range [IQR]). Categorical variables are presented as number (%).
Abbreviations: ICU, intensive care unit; LOS, length of stay

Supplementary Table 6. Independent risk factors for weaning failure after tracheostomy patients excluding those with neuromuscular diseases

Variable	Adjusted		
	OR	95% CI	*P*
**Age**	0.993	0.978 - 1.009	0.381
**Female sex**	1.155	0.723 - 1.845	0.547
**BMI (kg/m2)**	0.971	0.936 - 1.007	0.110
**CCI**	0.988	0.882 - 1.107	0.831
**Malignancy**	1.793	1.117 - 2.877	0.016
**CAD**	1.576	0.883 - 2.811	2.811
**APACHE II**	1.012	0.984 - 1.042	0.396
**SOFA**	0.982	0.920 - 1.048	0.586
**NT-proBNP > 3,271 pg/mL**	2.914	1.833 - 4.633	<0.001
**Early tracheostomy (≤ 10 days)**	0.553	0.349 - 0.877	0.012

Note: Analyzed using multivariate logistic regression
Abbreviations: BMI, body-mass index; CCI, charlson comorbidity index; CAD, coronary artery disease; APACHE, the acute physiology and chronic health evaluation; SOFA, sequential organ failure assessment; NT-proBNP, N-terminal pro-B-type Natriuretic Peptide
