# Supplementary figures and images for: Admission NT-proBNP as a Prognostic Biomarker for Ventilator Weaning Failure: Implications for Tracheostomy Timing
Source: Biomedicines. 2026 Apr 17;14(4):916. doi: 10.3390/biomedicines14040916 (PMC13113426; doi:10.3390/biomedicines14040916)

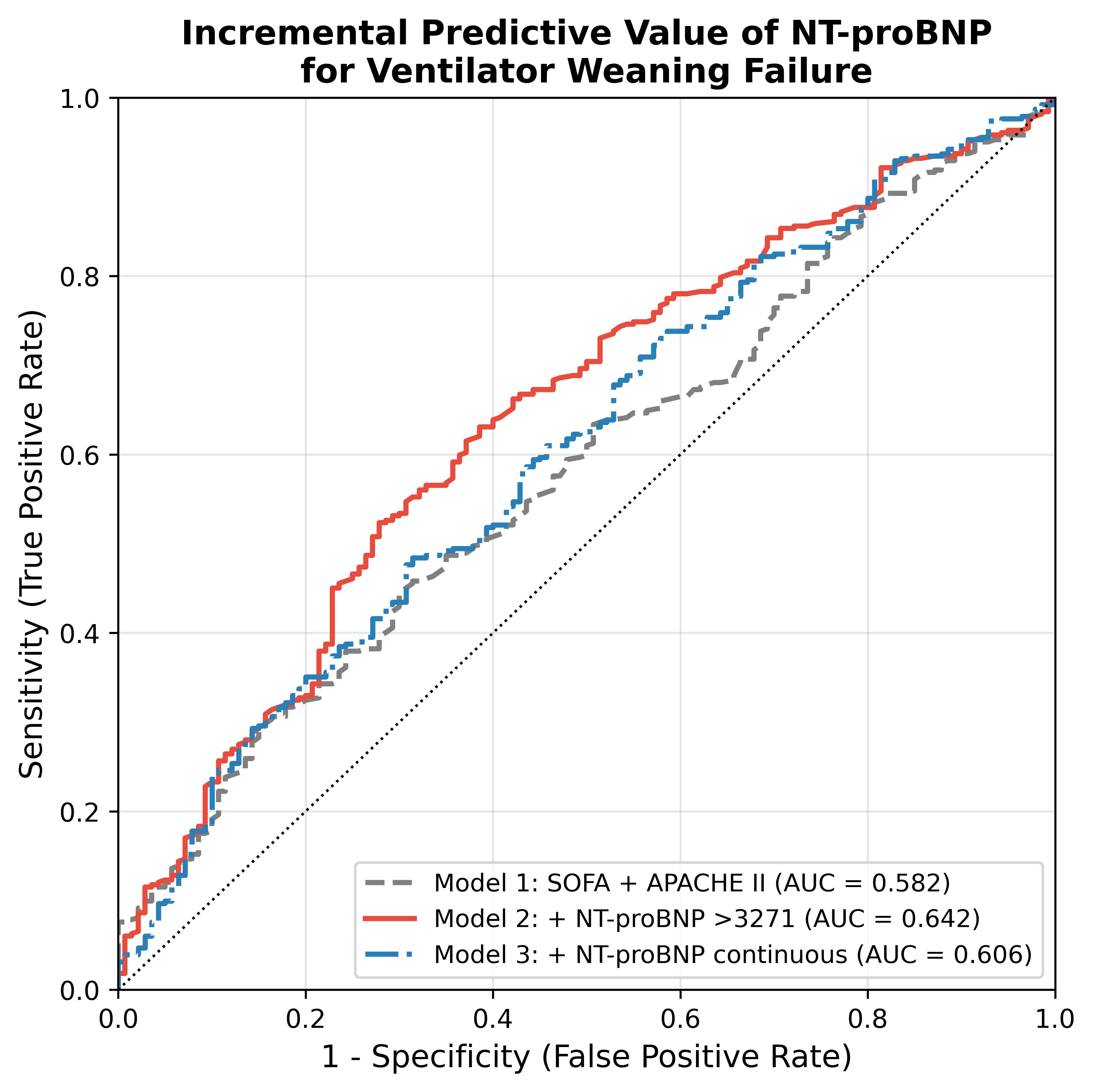

Supplement: Supplementary file 1 [file biomedicines-14-00916-s001.zip › Supplementary Figure 1.tiff]
